# Gremlin in the pathogenesis of hepatocellular carcinoma complicating chronic hepatitis C: an immunohistochemical and PCR study of human liver biopsies

**DOI:** 10.1186/1756-0500-5-390

**Published:** 2012-07-29

**Authors:** Maha Guimei, Nahed Baddour, Dalal ElKaffash, Laila Abdou, Yousry Taher

**Affiliations:** 1 Departments of Pathology, Champillion street, Alexandria, Egypt; 2 Clinical Pathology, Champillion street, Alexandria, Egypt; 3 Alexandria Center for Women’s Health, Champillion street, Alexandria, Egypt; 4 Internal Medicine (Hepatobiliary unit), Champillion street, Alexandria, Egypt; 5 Faculty of Medicine, University of Alexandria, Azarita, PO Box 31211, Alexandria, Egypt

**Keywords:** Gremlin, Bone morphogenetic protein 7, Hepatocellular carcinoma, Hepatic progenitor cells

## Abstract

**Background:**

The possible role of secretory products of fibrous tissue in the development of hepatocellular carcinoma (HCC) complicating chronic hepatitis C was investigated. Our hypothesis was that gremlin, secreted by fibroblasts, inhibited bone morphogenic protein (BMP), which mediates stem cell maturation into adult functioning hepatocytes, and thus, arrest stem cell maturation and promoted their proliferation in an immature state possibly culminating into development of HCCs.

**Results:**

Protein expression of cytokeratin 19 (CK19) and fibroblast growth factor 2 (FGF-2), and mRNA expression of gremlin and BMP-7 were studied in 35 cases of chronic hepatitis, cirrhosis and HCC complicating chronic hepatitis C. CK19 expression was higher in cases of cirrhosis (0.004), which correlated with the grade (r = 0.64, p = 0.009) and stage (r = 0.71, p = 0.001). All HCCs were negative for CK19. Stem cell niche activation (as indicated as a ductular reaction) was highest in cases of cirrhosis (p = 0.001) and correlated with CK19 expression (r = 0.42, p = 0.012), the grade(r = 0.56, p = 0.024) and stage (0.66, p = 0.006). FGF-2 expression was highest in HCCs and correlated with the grade (r = 0.6, p = 0.013), stage (0.72, p = 0.002), CK19 expression (r = 0.71, p = 002) and ductular reaction (0.68, p = 0.004) in hepatitis cases. Higher numbers of cirrhosis cases and HCCs (p = 0.009) showed gremlin expression, which correlated with the stage (r = 0.7, p = 0.002). Gremlin expression correlated with that of CK19 (r = 0.699, p = 0.003) and FGF2 (r = 0.75, p = 0.001) in hepatitis cases.

**Conclusions:**

Fibrosis promotes carcinogenesis by fibroblast-secreted gremlin that blocks BMP function and promotes stem cell activation and proliferation as well as possibly HCC development.

## Background

The variability in the prognosis of individuals with hepatocellular carcinoma (HCC) suggests that HCC may comprise several distinct biological phenotypes. These phenotypes may result from activation of different oncogenic pathways during tumorigenesis and/or a different cell of origin [1]. Enhanced proliferation and maturation arrest of hepatic progenitor/stem cells have been considered the origin of HCCs [1]. However, the link between cirrhosis and stem cell proliferation has not been fully elucidated. This link is multifaceted and complex with interplay of multiple factors that enhance or inhibit the proliferative rate of hepatic progenitor/stem cells, which culminates in HCC development. The outcome depends on the balance between stimulatory and regulatory signals that reach these stem cells [2,3]. The fate of hepatic progenitor/stem cells is determined by various growth factors, some of which are promoters of stem cell differentiation, and their exit from the stem cell niche as bone morphogenic proteins (BMPs), whereas other growth factors, such as basic fibroblast growth factor (b- FGF) and activin/nodal (the other arm of action of Transforming growth factor β (TGF-β) maintain their stemness and promote pluripotency [4]. During embryogenesis, BMP-7 mediates sprouting of the liver bud from the central foregut endoderm, which is essential for hepatocytic differentiation [4]. BMP-7 protein has been found in inflamed fibrotic livers [5] and its mRNA has been detected in stellate and Kupffer cells of the adult liver [6]. Several proteins antagonize BMP signaling, such as follistatin, noggin and members of the DAN family including cerberus and gremlin [7]. Gremlin is a protein secreted by fibroblasts and its mRNA is highly expressed under fibrotic conditions such as advanced diabetic nephropathy [8].

In this study, we presumed that gremlin, secreted by hepatic myofibroblasts in cases of fibrosis (cirrhosis), would antagonize the effects of BMP on stem cell differentiation and its anti-fibrogenic property. Antagonism of BMP-7 may arrest stem cell maturation and promote proliferation in an immature state, thereby possibly culminating in HCC development.

We investigated the role of cirrhosis and FGF-2 in the pathogenesis of HCC development complicating chronic HCV by detection of gremlin and BMP-7 mRNA and correlating their presence with hepatic progenitor cell numbers in cases of cirrhosis and HCC complicating chronic HCV in Egyptian patients with HCV type 4.

## Results

Patient ages ranged from 23 to 52 (mean ± standard deviation (SD) 36 ± 8.7), and 39 to 70 (mean ± SD 53.67 ± 7.4) for hepatitis, and cirrhosis and HCC cases, respectively (cirrhosis and HCC cases were obtained from the same segmentectomy specimens).

### Histopathological studies

Grades of the hepatitis group ranged from 1 to 7 (mean ± SD 2.93 ± 1.6), in which none were grade 0, seven (18.8%) each were grades 1 or 2, 13 (37.5%) were grade 3, two (6.3%) were grade 4, four (12.5%) were grade 5, none were grade 6, and two (6.3%) were grade 7 (Additional file 1: Table S1). While those of the cirrhosis group ranged from 1 to 8 (mean ± SD 4.3 ± 2.5), in which six (17%) were grade 1, two (6.7%) were grade 2, four (13.3%) were grade 3, eight (22%) were grade 4, none were grade 5, and five (14%) each were grades 6, 7 or 8 (Additional file 1: Table S1). Edmondson’s grades of HCCs were three cases of grade 1, 14 cases of grade 2, nine cases of grade 3 and nine cases of grade 4. While no differences were observed between the histological grades of hepatitis and cirrhosis cases (p = 0.09), significant differences in the ductular reaction (p = 0.001) were observed between these two groups (Table 1). The ductular reaction (Additional file 2: Figure S1) correlated with the grade (r = 0.56, p = 0.024) and stage (0.66, p = 0.006) in hepatitis cases.

**Table 1:** Scores of ductular reactions among groups

**Marker**	**Hepatitis**	**Cirrhosis**	**HCC**	
	**(n=35)**	**(n=35)**	**(n=35)**	
**Ductular reaction**	**n(%)**	**n(%)**		
**0**	7(18%)	0(0.0%)	NA	**MCp 0.001**
**1**	19(56%)	0(0.0%)	NA	
**2**	7(18%)	8(22.9%)	NA	
**3**	2(6%)	27(77.1%)	NA	

*NA* not assayed.

*MCp* p value for the Monte Carlo test.

### Immunohistochemical results

**Figure 1:**
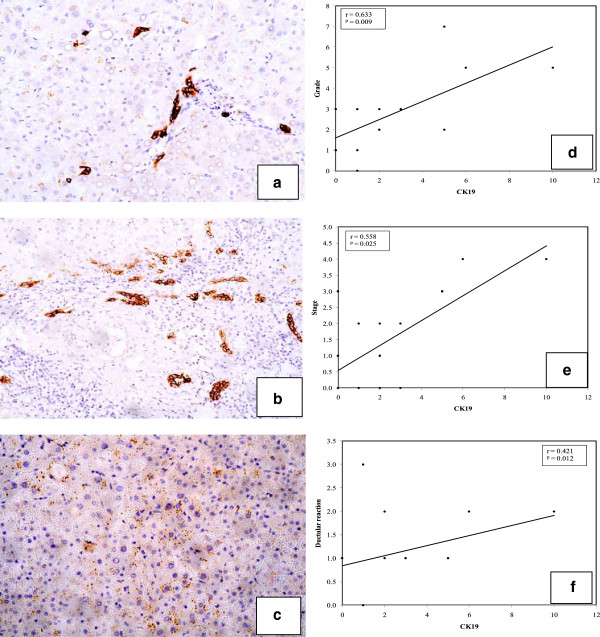
**Cases of hepatitis, cirrhosis and HCC stained for CK19.** A case of chronic hepatitis showing (**a**) single scattered CK19-positive cells in the lobule (arrow) (×100). (**b**) A case of cirrhosis showing a higher grade of CK19-positive ductular reaction and individual positive cells in the lobule (arrow). (CK19, ×100). (**c**) A case of HCC showing negativity for CK19 in tumor cells. (Streptavidin-peroxidase technique, anti-CK19 monoclonal antibody, ×200)*.***In right panels**: Line graphs show significant correlations between CK19 and each (**d**) grade, (**e**) stage and (**f**) ductular reaction.

Positive staining of CK19 in cell membranes was observed in both hepatitis and cirrhosis groups. Positivity was observed in and around portal tracts and along the fibrous septa with few intra-acinar positive cells (Figure 1a, b and c). Values were in the range of 0–10 (× 2.63 ± 2.7) and 0–90 (× 18 ± 23.48) for hepatitis and cirrhosis cases, respectively. Cirrhosis cases (Figure 1b) showed higher levels of positivity (p = 0.004) than hepatitis cases (Figure 1). All HCC cases (Figure 1c) were negative for CK19 (Table 2).

**Table 2:** CK19 positivity among groups

**CK 19**	**Hepatitis**	**Cirrhosis**	**HCC**
	**(n=35)**	**(n=35)**	**(n=35)**
**Range**	0.0 – 10.0	0.0 – 90.0	0.0 – 0.0
**Mean ± SD**	2.63 ± 2.70	18.0 ± 23.48	0.0 ± 0.0
**Median**	2.0	9.0	0.0
**Z**_**1**_**(p)**		2.866^*^ (0.004)	4.305^*^ (<0.001)
**Z**_**2**_**(p)**			4.665^*^ (<0.001)

*Z*_*1*_ Z for the Mann–Whitney test between hepatitis and other groups.

*Z*_*2*_ Z for the Mann–Whitney test between cirrhosis and HCC groups.

*Statistically significant.

Among hepatitis cases, positive correlations were observed between each of CK19 expression and each grade (Figure 1d) and stage (Figure 1e) (r = 0.63, p = 0.009 and r = 0.56, p = 0.025, respectively). Correlation with the stage was not found in cirrhosis cases (fixed stage 6 Hepatitis activity index (HAI) by definition). However, correlation with the grade was detected (r = 0.753, p = 0.001). Ductular reactions correlated with CK19 expression in hepatitis cases (r = 0.42, p = 0.012) (Figure 1f).

**Figure 2:**
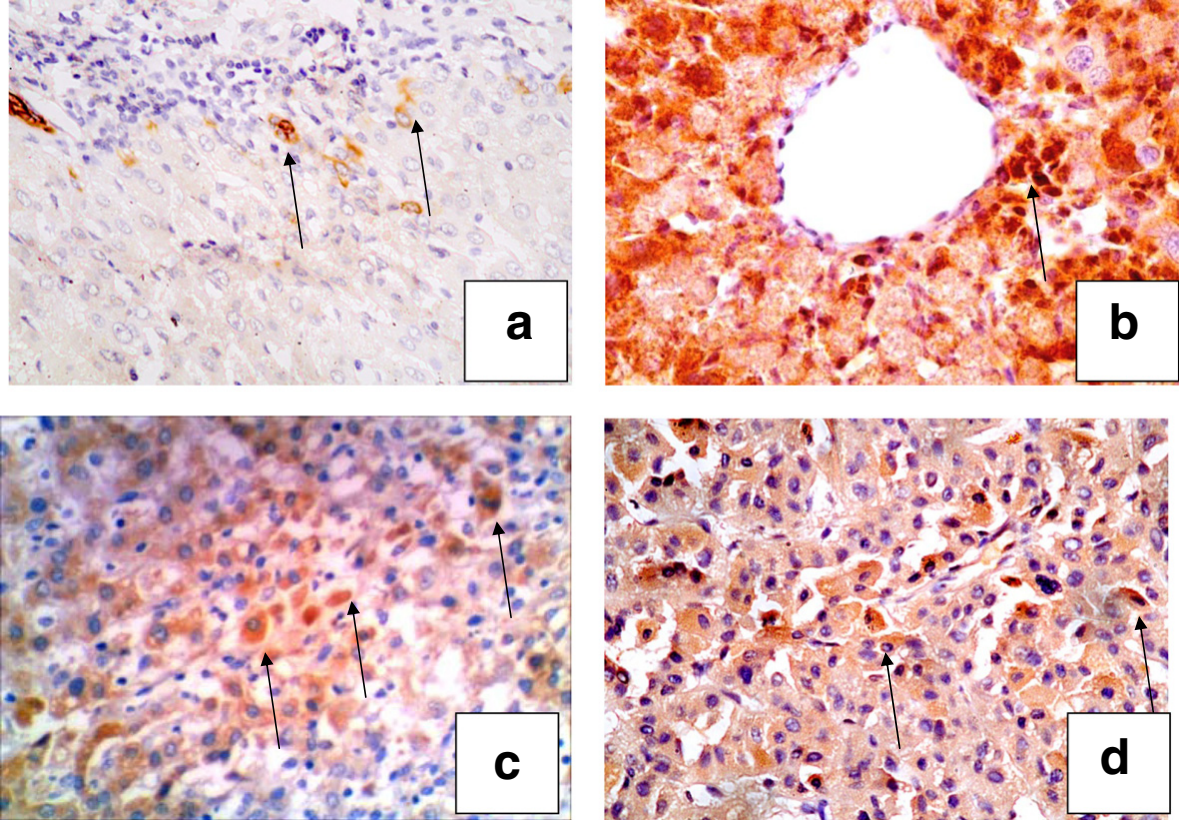
**Cases of hepatitis, cirrhosis and HCC stained for FGF-2.** A case of chronic hepatitis showing a few positive hepatocytes in (**a**) periportal areas (×200), (**b**) the pericentral zone (×400) and (**c**) intermediate hepatocytes (arrow) (×200). In HCC, (**d**) positivity was also detected in HCC tumor cells (×200). (streptavidin-peroxidase technique, anti-FGF2 monoclonal antibody).

Hepatitis and cirrhosis groups showed positive FGF-2 expression as granular cytoplasmic staining in periportal (Figure 2a), perivenular (Figure 2b) and intermediate (Figure 2c) hepatocytes as well as bile ductular epithelial cells. In HCCs, malignant hepatocytes also expressed FGF-2 (Figure 2d). FGF-2 positivity was higher in HCCs than in hepatitic and cirrhotic tissues, but was not significant (Table 3).

**Table 3:** Level of FGF-2 positivity among groups

**FGF 2**	**Hepatitis**	**Cirrhosis**	**HCC**
	**(n=35)**	**(n=35)**	**(n=35)**
**Range**	0.060.0 – 16.0.0	0. 0.0 – 16.00 – 16.0	0.0 – 70.0
**Mean ± SD**	5.06 ± 4.30± 4.30	6.13 ± 5.0.0	14.67 ± 18.67
**Median**	4.544.50	4.04.0	9.0
**Z1 (p)**		.50.557(0.578)	1.308 (0.191)
**Z2 (P)**			0.937 (0.349)

*Z*_*1*_ Z for the Mann–Whitney test between HCV and other groups.

*Z*_*2*_ Z for Mann Whitney test between cirrhosis and HCC.

* Statistically significant at p ≤ 0.05.

**Figure 3:**
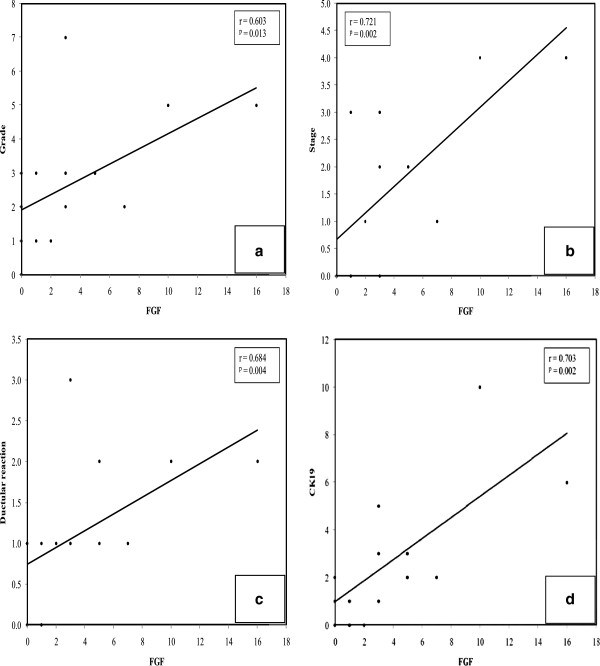
Line graphs of correlations between FGF-2 positivity and each (a) grade, (b) stage, (c) ductular reaction and (d) CK19 positivity.

FGF-2 correlated with each of grade (r = 0.603, p = 0.013) (Figure 3a, Table 4), stage (r = 0.721, p = 0.002) (Figure 3b, Table 4) and ductular reaction (r = 0.684, p = 0.004) (Figure 3c, Table 4) in hepatitis cases. In hepatitis and cirrhosis cases, positive correlations were detected between FGF-2 and CK19 positivity (r = 0.703, p = 0.002 (Figure 3d) and r = 0.536, p = 0.048, respectively) (Table 4).

**Table 4:** Correlations between FGF-2 and other parameters among groups

**FGF-2**	**Grade**	**Stage**	**Ductular reaction**	**CK19**
**HCV r**	0.603^*^	0.721^*^	0.684^*^	0.703^*^
**patients p**	**0.013**	**0.002**	**0.004**	**0.002**
**Cirrhotic r**	0.446	0.388	0.483	0.536*
**patients p**	0.096	0.153	0.080	**0.048**

*Rho* spearman’s correlation coefficient.

*p < 0.05.

### Gremlin expression and its correlations

**Figure 4:**
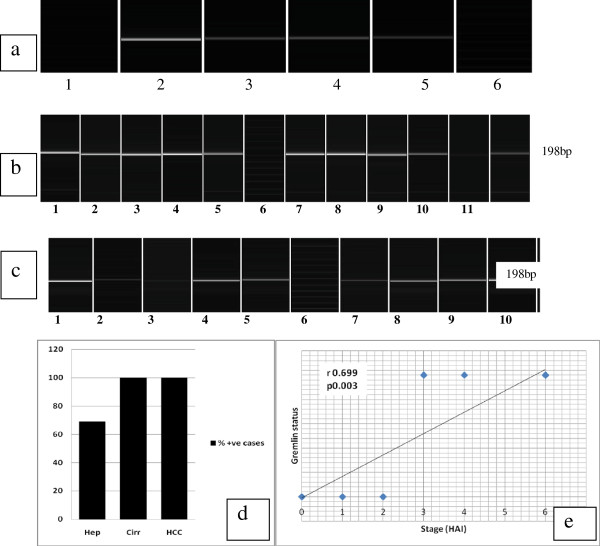
**Gremlin expression among the three study groups.** (**a**) Gremlin mRNA expression in HCV cases. PCR products are observed in lanes 2, 3, 4 and 5. PCR products are not observed in lane 1. A 25 bp size marker was used in lane 6. Note: additional bands. (**b**) cirrhosis specimens. Note: all cases show a positive band at 198 bp. Lane 6 is a 25 bp marker (**c**) In HCC specimens, all cases show a positive band at 198 bp. Lane 6 is a 25 bp marker (Capillary electrophoresis using e-gene*,* HAD-GT12). (**d**) Level of gremlin positivity among study groups. (**e**) Significant correlation between the stage and gremlin expression.

Gremlin mRNA (198 bp) was expressed in 24/35 (68.7%) hepatitis cases (Figure 4a), 35/35 (100%) cirrhosis cases (Figure 4b), and 35/35 (100%) HCC cases (Figure 4c) (Table 5, Figure 4d). Gremlin mRNA levels were higher in HCCs and cirrhosis cases than in hepatitis cases (p = 0.009) (Table 5, Figure 4d).

**Table 5:** Levels of positivity for gremlin and BMP-7 among groups

**Positive cases (%)**	**Hepatitis**	**Cirrhosis**	**HCC**	**MCp**
	**n (%)**	**n (%)**	**n (%)**	
**Gremlin mRNA**	24 (68.7%)	35 (100%)	35 (100%)	0.009*
**BMP 7 mRNA**	16 (43.8%)	27 (78.6%)	25 (73.3%)	0.115

*n* actual numbers of positive cases.

*MCp* p value for the Monte Carlo test.

*Statistically significant at p ≤ 0.05.

In hepatitis cases, gremlin expression correlated with the stage (r = 0.699, p = 0.003) (Figure 4e) and CK19 positivity (r = 0.223, p = 0.04) (Table 6), but not with the stage in the cirrhosis group due to the fixed stage (stage 6 HAI by definition). Gremlin expression did not correlate with the number of FGF-2 positive cells (r = 0.548, p = 0.191) or BMP-7 mRNA level (r = 0.051, p = 0.851) in the hepatitis group. However, it did positively correlate with BMP-7 levels in the cirrhosis group (r = 0.602, p = 0.023) (Table 6).

**Table 6:** Correlations between gremlin mRNA expression and studied markers in hepatitis and cirrhosis groups

**Gremlin**	**Grade**	**Stage**	**Ductular reaction**	**CK 19**	**BMP 7**
**HCV r**	0.136	0.699^*^	0.276	**0.223**	0.051
**p**	0.615	**0.003**	0.301	**0.04**	0.851
**Cirrhosis**					
**r**	**0.526**		**0.994**		**0.602**
**p**	**0.044**	NP	**0.001**	NP	**0.023**

*Rho (r)* Spearman coefficient *Statistically significant at p ≤ 0.05.

*NP* Not performed because gremlin was expressed in 100% of cases and all 35 cases of this group received a score of 3 for CK19 positivity. Therefore, a correlation was not feasible.

Note: a correlation was not performed with the stage (fixed stage 6 by definition).

### BMP-7 expression and its correlations

**Figure 5:**
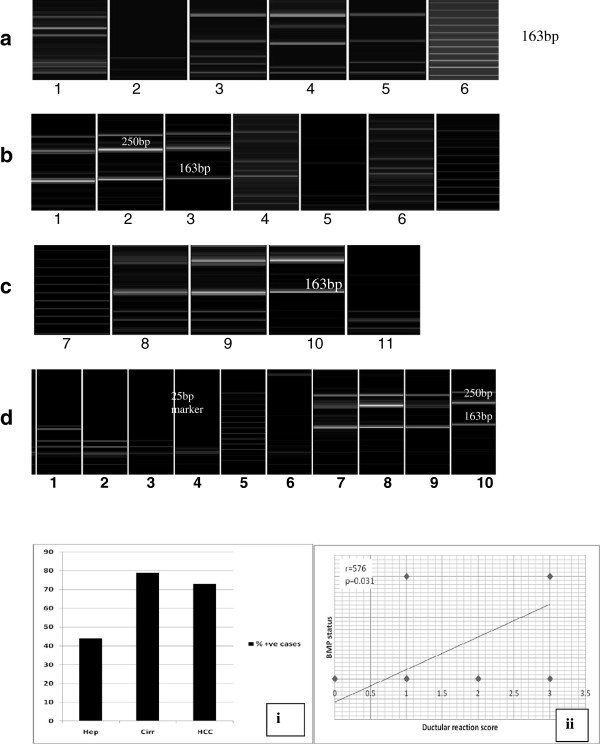
**Gel electrophoreograms of BMP expression among study groups.** (**a**) BMP-7 mRNA expression in liver tissue from patients with chronic hepatitis C. BMP-7 expression is observed in lanes 1, 3, 4 and 5 at 163 bp, whereas no DNA bands are present in lane 2. Note the extra higher band at 250 bp. Lane 6 is a 25 bp size marker. (**b**): BMP-7 mRNA expression in cirrhotic liver tissue. PCR products are observed at 163 bp in lanes 1, 2, 3, 4 and 6. Lane 5 shows no PCR products. Lane 7 is a 25 bp size marker. (**c**): BMP-7 mRNA expression in cirrhotic liver tissue. PCR products are observed in lanes 8, 9 and 10, but not in lane 11. Lane 7 contains the marker. (**d**): BMP-7 mRNA expression in HCC cases. PCR products are observed in lanes 1, 7, 8, 9 and 10. No PCR products are observed in lanes 2, 3, 4 and 6. Lane 5 contains the marker. (Capillary electrophoresis using e-gene, HAD-GT12). **(i)** Bar graph showing the numbers of cases expressing BMP-7 mRNA among study groups. **(ii)** Correlations between BMP-7 mRNA expression and ductular reactions in the cirrhosis group.

No significant differences in the level of BMP-7 mRNA expression between the three study groups were detected (Figure 5a–d and i, Table 5). However, BMP-7 mRNA correlated significantly with the levels of ductular reactions (r = 0.576, p = 0.031) in the cirrhosis group (Figure 5ii) (Table 7).

**Table 7:** Correlations between BMP-7 mRNA levels and studied markers among groups

**BMP 7**	**Grade**	**Stage**	**Ductular reaction**	**CK 19**	**FGF 2**	**Gremlin**
**HCV**						
**r**	−0.311	0.217	0.076	0.264	0.136	0.051
**p**	0.241	0.418	0.780	0.324	0.608	0.851
**Cirrhosis**						
**r**	0.284	0.134	0.576^*^	0.065	0.196	0.602*
**p**	0.326	0.635	**0.031**	0.825	0.501	**0.023**

*Rho (r)*: Spearman coefficient.

*Statistically significant at p ≤ 0.05.

## Discussion

The current study aimed to identify a possible link between fibrosis/cirrhosis and HCC development in chronic HCV type 4 cases. Under normal conditions, FGFs, acting via BMP-7, stimulate maturation of stem cells into functional mature hepatocytes or cholangiocytes. However, under fibrotic conditions, gremlin is produced by fibrotic tissue [7,8] and inhibits BMP function [7,8]. This production of gremlin may cause maturation arrest of stem cells and their proliferation in an immature state predisposes HCC development.

We found that CK19 was expressed at higher levels in cirrhosis cases, compared with those of hepatitis cases. CK19 expression correlated with the grade and stage of the disease. These findings are consistent with data from Ravazoula et al [9] and Libbrecht et al [10]. The correlation between stem cell niche activation, manifested as significantly increased ductular reactions [11], and increased severity (grade/stage/fibrosis) of the disease, which were observed in the present study, confirms the notion that damage of the epithelial compartment of the liver by chronic disease causes replicative senescence of hepatocytes, which leads to reserve cell compartment activation [12]. Several studies using detailed immunophenotyping of HCCs show that a substantial number of HCCs (28–50%) express progenitor cell markers including CK7, CK19 and OV6 [13-15]. In the present study, although significantly higher numbers of CK19-positive cells were observed in cirrhosis cases, HCCs did not show CK19 positivity. This observation can be interpreted in two ways: first, expression of immune markers by stem cells is stage-specific and not lineage-specific [16]; and CK19 represents an early marker of stem cells, which is normally expressed in hepatoblasts [17]. In HCCs and chronic HCV type 4 cases, stem cells are probably at a different stage of maturation/differentiation than that required to express this particular marker. Alternatively, the negativity of our HCC cases for CK19 may be attributed to the clinical setting in which our study was conducted. Other studies have been performed using experimental models of carcinogenesis [18], or a different disease setting (etiologic factors other than HCV type 4) such as the studies by Durnez et al [15], Lyer et al [18] and others [16] where the etiology of liver disease was quite heterogeneous with only very few cases resulting from HCV infection. Our study was conducted exclusively with HCV type 4 induced HCCs; the main predisposing factor of HCC in Egypt [19]. The pathogenetic pathways activated in HCCs complicating HCV may be quite different from those occurring in other settings such as chemical carcinogenesis. In fact, even chemical carcinogenic regimens result in activation of different pathways. Lingala et al [20] show similar results using a different set of stem cell markers (CD44, CD90 and CD133) with few HCCs showing very limited expression of these markers, although they were found in cases of chronic HCV and dysplastic foci. Dunsford et al [21] used monoclonal antibodies to study sequential histopathologic changes that occurred during two regimens of chemical carcinogenesis in the rat. The Solt-Farber regimen caused prominent oval cell proliferation and multiple large dysplastic nodules, while continuous administration of diethylnitrosamine produced minimal oval cell proliferation and a few small nodules. These results demonstrate that different etiologies lead to HCC development via different pathogenetic pathways. Is it possible that different cancer stem cells are activated by different pathogenetic pathways, which results in a tumor (HCC) that is actually not a single entity, but biologically different tumors with a common morphology [22,23].

Gremlin antagonizes BMP function under several disease conditions including idiopathic pulmonary fibrosis [24], proliferative vitreoretinopathy [25] and renal fibrosis [26]. It non-covalently binds to BMP family members to prevent interactions with their cognate receptors, thus altering the effective concentration of active signalling molecule [27]. There are also claims that gremlin possesses an oncogenic role. Data from Namkoong et al [28] and Sneddon et al [29] have shown that gremlin is over-expressed in various human malignancies including carcinomas of the cervix, lung, ovary, kidney, breast, colon and pancreas as well as sarcomas. The source of gremlin in highly cellular HCCs is Kupffer cells [30]. In the present study, all three groups of patients expressed gremlin mRNA that was significantly higher in cirrhosis cases than hepatitis cases. Furthermore, gremlin mRNA significantly correlated with the stage. This finding may support the theory that fibroblast-secreted gremlin blocks BMP-mediated stem cell maturation [31], with possible HCC development in cirrhosis patients.

We detected FGF-2 expression in all study groups, with a significant correlation noted between FGF-2 positivity and each grade and stage. FGF-2 is considered as an autocrine repair hormone secreted following injury to promote healing (fibrosis) [32,33] and neoangiogenesis [34]. In our study, the distribution pattern of FGF-2 positivity in hepatitis cases is consistent with data from Xiaodong et al [35] who recently elucidated the localization of FGF-2 mRNA in these same regions in a mouse fibrotic liver model [35]. Furthermore, FGF-2 expression significantly correlated with CK19 expression, implying that promotion of fibrogenesis by FGF [36] leads to increased gremlin expression that blocks BMP-7-mediated stem cell differentiation, which leads to proliferation in an immature state, as shown by higher CK19 positivity. This explanation is further supported by our finding of a positive correlation between gremlin mRNA expression, FGF-2 and CK19 positivity in the hepatitis group. Thus, FGF-2 may have an oncogenic role in HCC development.

## Conclusions

Gremlin participates in the pathogenesis of HCC complicating chronic hepatitis C type 4-related cirrhosis by antagonizing the maturation inducing function of BMP-7 rather than the actual production of BMP-7 in cirrhotic livers. This occurrence promotes the proliferation of stem cells in an immature state, which may contribute to HCC carcinogenesis.

## Methods

### Patients

The present study was conducted using 35 liver core biopsies from patients with hepatitis (HCV type 4, shown as positive by PCR) and 35 freshly collected segmentectomy specimens from patients with HCC complicating HCV-related cirrhosis (segmentectomy specimens were the source for both cirrhotic and carcinoma tissues). Liver core biopsies were clinically indicated as a part of the treatment strategy for chronic HCV infection (baseline biopsy for grading and staging before combinatorial treatment with Interferon alpha 2 B (IFN-α2b) and ribavirin). The protocol of this study was approved by the Ethics committee of the Faculty of Medicine, University of Alexandria. Informed consent was obtained from patients for use of these specimens for research purposes.

Paraffin blocks of liver biopsies were obtained from the archives of the Pathology Department at the Alexandria Faculty of Medicine. Patients were selected sequentially from our archives for hepatitis cases and cases sequentially obtained from the clinic for cirrhosis and HCC cases (paired specimens). All HCC cases were complicating HCV type 4-related cirrhosis. Inclusion criteria were HCV type 4 (as shown by PCR using Inno-LiPA I and II) patients, and exclusion criteria included anti- hepatitis B virus surface antibody HBV positivity, obesity, history of alcoholism and diabetes mellitus.

### Histopathologic study

Hematoxylin-eosin sections were examined to determine the grade and stage of the hepatitis and cirrhosis cases [37], ductular reaction and HCC grade [38]. Scores were the mean determinations. A ductular reaction, a sign of stem cell niche activation, was graded on a scale from 0 to 3, according to the number of points of origin in the perimeter of portal tracts showing a ductular reaction, as follows: 0, none; 1, two or less points; 2, two to four points; and 3, > four points.

### Immunohistochemical staining

Antibodies against CK19 (1:100, clone A53-B/A2.26).

Lab Vision Corporation, Fremont, CA, USA), a stem cell marker, and FGF-2 (1:50, clone Ab16828; Abcam, Cambridge, UK were used together with an Ultravision LP detection System, (Thermo scientific, Lab vision, catalog # TL-015HD, Fremont, CA) horse radish peroxidase polymer (HRP) polymer and diaminobenzidine (DAB) plus chromogen (Cat#: TL-015HD; Lab Vision Corporation) according to the manufacturer’s instructions. Epitope retrieval was performed by boiling in 2% citrate buffer (10mM, pH 6.0) in a microwave at 700 W for 15 min. Sections were incubated with antibodies overnight at 4°C. Bile ducts served as internal positive controls for CK19 and kidney tubules for FGF-2. A positive reaction was considered only if single cells (not forming part of a ductular reaction) showed membrane staining for CK19 and cytoplasmic staining for FGF-2. Positive cells were counted in 10 random high power fields (×400) for each case by two pathologists, separately. Results were presented as the mean ± SD of both readings.

### RNA extraction and reverse transcription PCR analysis

Total RNA was extracted from fresh snap frozen liver tissue using Trizol (Cat# 15596-026, Invitrogen; Madison, WI, USA). The concentration and purity of extracted RNA were determined using a nanodrop ND-1000 spectrophotometer. Samples were accepted when the A260/A280 ratio (nucleic acid/protein absorbance) was ≥ 1.8. Reverse transcription of extracted RNA was performed using random hexamer primers and a Transcriptor First Strand cDNA Synthesis kit (Cat#: 04 379 012 001; Roche diagnostics, Switzerland) according to manufacturer’s instructions. cDNA was either processed immediately or stored at –20°C. cDNA amplification was performed with a QB-96 thermal cycler using Atlas hotTaq (Lot#: Js20080508; Bioatlas, Tartu, Estonia) under the following conditions: 15 min at 95°C followed by 40 cycles of 45 s at 92°C, 30 s at 55°C and 90 s at 72°C. Primer sequences were those described by Wordinger et al [39]. Gene detection was performed by e-gene capillary electrophoresis, HAD-GT12 according to the manufacturer’s instructions. The housekeeping gene, PBGD was used to confirm the integrity of extracted RNA.

BMP-7; F: AGCCCGGGTAGCGCGTAGAG, R: GCGCCGGTGGATGAAGCTCGA; gremlin (DRM); F: ATCAACCGCTTCTGTTACGG, R: ATGCAACGACACTGCTTCAC. (Cat#s. 10336-022; D4063F04, for BMP-7 and D4063F05, D4063F06, D4063F07, for DRM; Invitrogen, Madison, WI, USA).

### Statistical analyses

Statistical analyses were performed using SPSS 17 (SPSS Inc., Chicago, IL, USA). Monte Carlo and Mann–Whitney tests were used to calculate the significance of differences between groups. Pearson and Spearman coefficients were used to find correlations between study parameters among patient groups.

## Competing interests

The authors have no competing interests to declare.

## Authors’ contributions

All authors have revised and critically appraised the manuscript and have approved its final version before sending it out seeking its publication. MG collected specimens and performed immunohistochemical staining, counted positively stained cells and collected references. DE conducted PCR. NB was responsible for study conception, immunostaining counts, correlations and writing the manuscript. YT provided specimens of tru cut liver core biopsies and LA supervised.

## Supplementary Material

Additional file 1 **Table S1.** Differences in the grade and stage among groups. Click here for file

Additional file 2 **Figure S1.** Ductular reactions among study groups. Note in (a) a portal tract with a ductular reaction from three points in its circumference (score of 2) (×200). (b) A case showing a ductular reaction originating from four points in the circumference of the tract (score of 2). (×400) (hematoxylin-eosin). Click here for file
