# Supplementary material for: Gremlin in the pathogenesis of hepatocellular carcinoma complicating chronic hepatitis C: an immunohistochemical and PCR study of human liver biopsies
Source: BMC Res Notes. 2012 Jul 29;5:390. doi: 10.1186/1756-0500-5-390 (PMC3506438; doi:10.1186/1756-0500-5-390)
Supplement: Additional file 1 — Table S1. Differences in the grade and stage among groups. [file 1756-0500-5-390-S1.doc]

**Additional file 1: Table S1. Differences in the grade and stage among** groups.

|  | **HCV** | | **Cirrhosis** | | **Test of sig.** |
| --- | --- | --- | --- | --- | --- |
|  | **No.** | **%** | **No.** | **%** |  |
| **Stage** |  |  |  |  | MCp <0.001^*^ |
| Stage 0 | 13 | 37 | 0 | 0.0 |  |
| Stage 1 | 4 | 11.5 | 0 | 0.0 |  |
| Stage 2 | 7 | 20 | 0 | 0.0 |  |
| Stage 3 | 7 | 20 | 0 | 0.0 |  |
| Stage 4 | 4 | 11.5 | 0 | 0.0 |  |
| Stage 6 | 0 | 0.0 | 35 | 100.0 |  |
| **Grade** |  |  |  |  |  |
| Grade 0 | 2 | 6.3 | 0 | 0.0 | MCp=0.091 |
| Grade 1 | 7 | 18.8 | 6 | 17.0 |  |
| Grade 2 | 7 | 18.8 | 2 | 6.7 |  |
| Grade 3 | 13 | 37.5 | 4 | 13.3 |  |
| Grade 4 | 0 | 0.0 | 8 | 22 |  |
| Grade 5 | 4 | 12.5 | 0 | 0.0 |  |
| Grade 6 | 0 | 0.0 | 5 | 14 |  |
| Grade 7 | 2 | 6.3 | 5 | 14 |  |
| Grade 8 | 0 | 0.0 | 5 | 14 |  |

MCp: p value of the Monte Carlo test
