# Supplementary figures and images for: Gremlin in the pathogenesis of hepatocellular carcinoma complicating chronic hepatitis C: an immunohistochemical and PCR study of human liver biopsies
Source: BMC Res Notes. 2012 Jul 29;5:390. doi: 10.1186/1756-0500-5-390 (PMC3506438; doi:10.1186/1756-0500-5-390)

**Additional file 2: Figure S1**: **Ductular** **reaction in the study cases.**


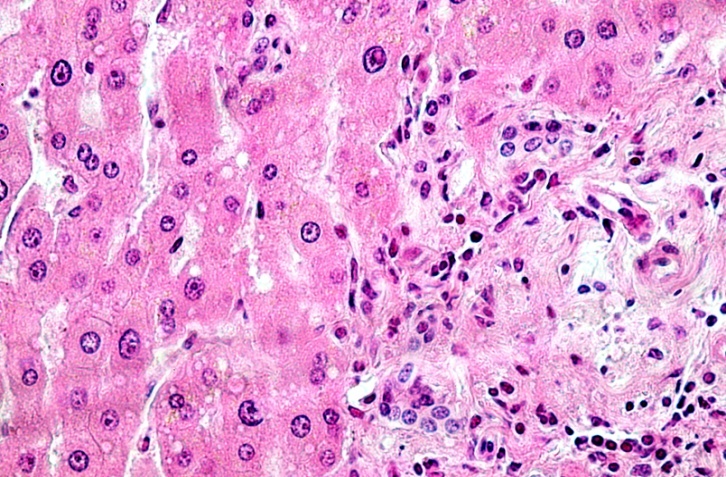


**a**


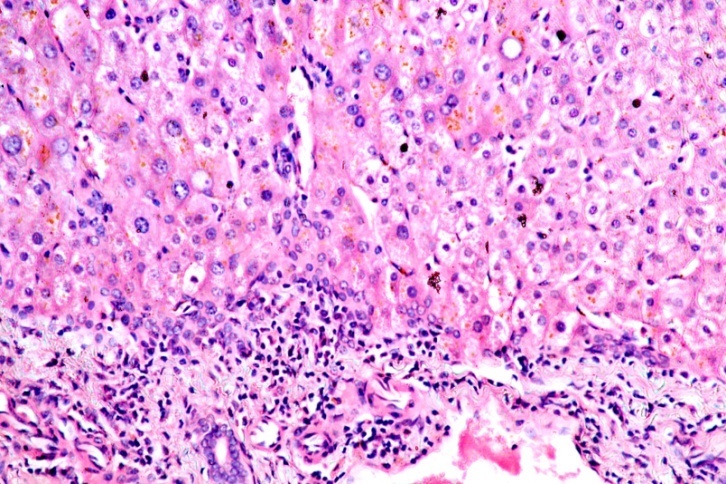


**bb**

Supplement: Additional file 2 — Figure S1. Ductular reactions among study groups. Note in (a) a portal tract with a ductular reaction from three points in its circumference (score of 2) (×200). (b) A case showing a ductular reaction originating from four points in the circumference of the tract (score of 2). (×400) (hematoxylin-eosin). [file 1756-0500-5-390-S2.docx]
